# Monomodular and multifunctional processive endocellulases: implications for swine nutrition and gut microbiome

**DOI:** 10.1186/s42523-024-00292-w

**Published:** 2024-02-02

**Authors:** Ming Z. Fan, Laurence Cheng, Min Wang, Jiali Chen, Wenyi Fan, Fatmira Jashari, Weijun Wang

**Affiliations:** 1 Department of Animal Biosciences, University of Guelph, N1G 2W1 Guelph, ON Canada; 2 One Health Institute, University of Guelph, N1G 2W1 Guelph, ON Canada; 3 Transpharmation LTD, N1M 2W3 Fergus, ON Canada; 4 Department of Human Health and Nutritional Sciences, University of Guelph, N1G 2W1 Guelph, ON Canada; 5 The Canadian Food Inspection Agency Ontario Operation, N1G 4S9 Guelph, ON Canada

**Keywords:** Biomass, Fibre enzyme, Gut microbiome, Gut permeability, Prebiotic, Pig

## Abstract

Poor efficiency of dietary fibre utilization not only limits global pork production profit margin but also adversely affects utilization of various dietary nutrients. Poor efficiency of dietary nutrient utilization further leads to excessive excretion of swine manure nutrients and results in environmental impacts of emission of major greenhouse gases (GHG), odor, nitrate leaching and surface-water eutrophication. Emission of the major GHG from intensive pork production contributes to global warming and deteriorates heat stress to pigs in tropical and sub-tropical swine production. Exogenous fibre enzymes of various microbial cellulases, hemicellulases and pectinases have been well studied and used in swine production as the non-nutritive gut modifier feed enzyme additives in the past over two decades. These research efforts have aimed to improve growth performance, nutrient utilization, intestinal fermentation as well as gut physiology, microbiome and health via complementing the porcine gut symbiotic microbial fibrolytic activities towards dietary fibre degradation. The widely reported exogenous fibre enzymes include the singular use of respective cellulases, hemicellulases and pectinases as well as their multienzyme cocktails. The currently applied exogenous fibre enzymes are largely limited by their inconsistent in vivo efficacy likely due to their less defined enzyme stability and limited biochemical property. More recently characterized monomodular, multifunctional and processive endoglucanases have the potential to be more efficaciously used as the next-generation designer fibre biocatalysts. These newly emerging multifunctional and processive endoglucanases have the potential to unleash dietary fibre sugar constituents as metabolic fuels and prebiotics, to optimize gut microbiome, to maintain gut permeability and to enhance performance in pigs under a challenged environment as well as to parallelly unlock biomass to manufacture biofuels and biomaterials.

## Introduction

**Fig. 1:**
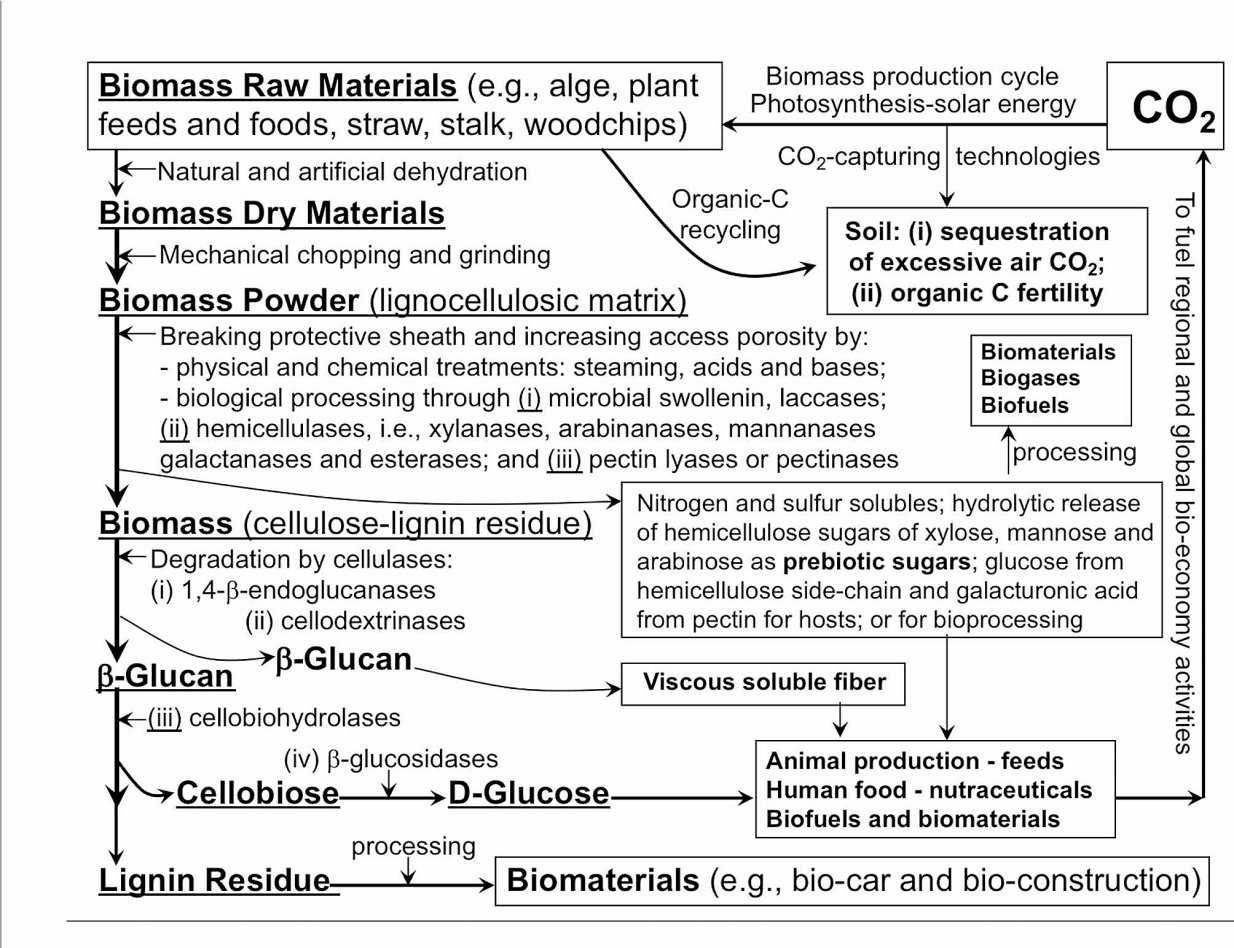
An illustration of the major processes and biological steps in utilization and conversion of plant biomass materials and dietary fibre in providing renewable functional ingredients to animal feed industry and human food industry as well as renewable lignocellulosic biofuels and biomaterials for a sustainable global bio-economy with an emphasis on the essential roles of the plant biomass and dietary fibre degradation microbial cellulases as novel industrial enzymes with adaption from Fan et al. [2]

Biomass materials, including cellulose, hemicelluloses, lignin and pectin, constitute dietary fibre components of plant and alge origins in animal and human nutrition. Biomass is produced at about 1.5-trillion dry metric tons yearly in the globe [1, 2]. Thus, for the long haul, global biomass replenishing production via recycling of the air-borne carbon dioxide (CO_2_), carried out by photosynthesis and driven by the solar energy, can potentially provide inexhaustible metabolizable and prebiotic sugars for nutrition, health promotion, food security and other basic organic monomer compounds for sustainable biofuels and biomaterials for mankind in development of the sustainable global bioeconomy, as further illustrated in Fig. 1.

Emission of the major greenhouse gases (GHG) has become the existential threat to the global community, e.g., the global warming-caused rising sea level and extreme weather patterns [3, 4]. The world agricultural food animal production accounts for 15–16% of the total human-induced GHG emission while pork production contributes at about 9% within the entire animal production sector [3, 4]. Global pork sector now represents about 35% of the overall meat production [5, 6]. As reviewed by Cheng et al. and Fan et al. [6, 7], the core global pork production economic sustainability issue is primarily attributed to the poor efficiency of dietary fibre utilization, which limits global pork production profit margin. Dietary fibre is well known as the anti-nutritive factors [6], thus the poor efficiency of dietary fibre utilization also adversely affects utilization efficiency of other dietary nutrients such as crude protein (CP), amino acid (AA), crude fat and minerals in swine. Furthermore, poor utilization efficiency of nutrients, including CP, AA and minerals, leads to some negative environmental sustainability concerns because of swine manure excessive excretion of carbon and nitrogenous compounds and various minerals [6]. These negative environmental issues include anaerobic biogenesis and emission of the potent major GHG of methane (CH_4_) and nitrous oxide (N_2_O) and volatile odor compounds from swine manure slurry storages as well as nitrate leaching and phosphorus run-off associated with swine manure [6]. Therefore, it is imperative to improve efficiency of dietary fibre utilization for the development of sustainable global pork production.

Because of the economic constraint, a significant proportion of the world swine production is operated under the challenged environmental conditions such as unsanitary barns for housing pigs under chronic heat stress in the summer months in the tropical and sub-tropical regions including some of the large-economy and very large swine production countries [8–10]. Increases in emission of the major GHG from intensive pork production further contribute to the global warming effect and further deteriorate heat stress to pigs under the tropical and sub-tropical swine production setting [9]. As reviewed by Cheng et al. and Fan et al. [6, 7], one important strategy for mitigation is to further develop efficacious exogenous fibre enzymes for the swine industry.

Here in this paper, we aim to conduct a critical reviewing of the following aspects: firstly, the current understanding of limiting factors affect biomass processing and dietary fibre utilization; secondly, we review the recent discovery and characterization of the newly emerged monomodular, multifunctional and processive endoglucanases; we also critically review the up-to-dated literature of efficacy, nutrition and gut physiological responses of currently existing exogenous fibre enzymes of various microbial cellulases, hemicellulases and pectinases that are applied for improving growth performance, nutrient utilization, intestinal fermentation as well as gut physiology, microbiome and health in pigs; and lastly we assess the limitations of the currently reported exogenous fibre enzymes and the potential applications of a new group of monomodular, multifunctional and processive designer cellulases to modulate growth performance, nutrient utilization, intestinal physiology and gut microbiome as novel microbial gut enzyme modifier feed additives and to engage in industrial biomass processing as newly emerging biocatalysts.

## Limiting factors in biomass and dietary fibre utilization

Biomass processing and dietary fibre utilization are commonly hindered by the plant cell wall recalcitrance that is collectively referring to the natural resistance of cell wall materials to microbial enzymatic deconstruction [2, 11]. The first layer of the biomass and fibre recalcitrance is reflected by its limited surface porosity diameter ranging 3.5–5.2 nm that is formed through cross-linking between the polyphenolic lignin and various hemicelluloses [2, 12]. Most microbial cellulases such as the bi-modular free cellulases and cellulosomes have an enzyme molecular size or dimension much larger than this upper porosity diameter size range (3.5–5.2 nm) that natural biomass and fibre materials possess, including various feedstuffs of the plant origin, thus limiting their accessibility to the hydrolytic cellulose surface area [2, 13]. The second layer of the natural plant cell wall recalcitrance is that natural cellulose polymers are further bundled up in a strong quasi-crystalline structure consisting of macrofibril, microfibril and elementary fibril units that are resistant to cellulosic enzyme degradation. As reviewed by Lynd et al. [13] and Fan et al. [2], most natural microbial cellulases such as free cellulases and cellulosomes have very limited and extremely low hydrolytic activities towards the natural crystalline cellulose in biomass materials and dietary fibre in comparison with hydrolytic activities towards other plant polymers such as starches and hemicelluloses.

As shown in Fig. 1, the first layer of the fibre biomass recalcitrance and protective sheath can be tackled via biological processing strategies, i.e., commercial application of various exogenous hemicellulases and pectinases under in vitro feed processing conditions and in vivo via dietary supplementation of exogenous fibre enzymes in swine nutrition, whereas use of lignin-degradation enzymes is very limited mainly due to the scarcity of lignin-degradation enzymes in commercial supplies and from the natural environment [2]. While plant feed material origins of the applicable prebiotics are the various hemicellulose-derived oligosaccharides, complete microbial hemicellulase enzymatic hydrolysis under in vivo and in vitro conditions would release their corresponding monosaccharides of xylose, mannose and arabinose as the typical monosaccharide prebiotic sugars for potentially promoting prebiotic bacterial proliferation (Fig. 1). The first layer of the fibre biomass recalcitrance can also be alternatively removed via chemical-physical processing, resulting in purified crystalline cellulose biomass without lignin. And lignin residuals can behave as a group of inhibitors to cellulases and hemicellulases in subsequent biomass processing steps and to endogenous digestive enzymes and exogenous enzymes in the digestive tracts of animals and humans [6]. Under this context, Solka-Floc is a typical commercially available pure crystalline cellulose product that is chemical-physical processed from economical biomass materials such as wheat straw and woodchips and this Solka-Floc cellulose has been widely used in nutrition research with applications for pigs and humans [14]. However, Solka-Floc is too expensive to be used at significant levels in commercial swine production. It is conceivable from the processed Solka-Floc cellulose example that in vitro chemical-physical processing of dietary fibre biomass materials for swine nutrition and production is likely limited in two major aspects, including 1) the high-processing cost and ii) the potential nutritive value loss of various hemicellulose-derived monosaccharide sugars as potential dietary prebiotic sugars that will be irreversibly lost and cannot be recovered from the pre-treatment alkaline and/or acidic solutions.

Furthermore, as shown in Fig. 1, the synergistical hydrolyses by the microbial cellulases of endocellulases and exo-cellulases and/or processive endocellulases would ensure the degradation of cellulose polymer into soluble cellodextrin including cellobiose without resulting in a significant amount of 1,4-ß-glucan as intermediate microbial hydrolytic products [2, 13]. And the final breakdown of cellodextrin into free glucose by microbial ß-glucosidases in the porcine gut lumen and in biomass processing is not considered to be a rate-limiting or bottleneck step [2, 13]. As illustrated in Fig. 1, ß-glucan, including 1,4-ß-glucan and branched-glucan, and pectin are regarded as the classic viscous soluble fibre components and are thus the well-recognized anti-nutritive factors in swine nutrition [6]. Thus, as reviewed by Lynd et al. [13] and Fan et al. [2], continued discovery and biological engineering of highly active and synergistical microbial cellulases of endocellulases and exo-cellulases, particularly very active processive endocellulases that hydrolyze natural crystalline cellulose, is the key to conquer recalcitrance in biomass material processing and efficient dietary fibre utilization. Without a doubt, the discovery and development of efficacious cellulases is a crucial and transformative area of research, considering its profound potential to resolve the global social, economic and environmental issues for sustainable development.

### Discovery of the porcine gut symbiotic bacterial multi-functional processive endocellulase

The digestive utilization of dietary fibre in pigs is, in part, contributed by gut microbiota and is regulated at the level of gut microbiome [15, 16] with insoluble fibre digestibility being relatively low and variable at about 23–60% [6, 17, 18]. Commercial exogenous fibre enzymes presently used in swine nutrition are primarily tailored from the light industrial and biofuel enzymes that are characterized and engineered from *Trichoderma*, *Humicola insolens* and *Aspergillus* fungal species and the *Bacillus* sp. with limited intrinsic enzyme structure and functionality specificity in porcine dietary fibre degradation [2, 7, 19]. The endpoint responses of in vivo efficacy of the current exogenous fibre enzymes are frequently inconsistent and at variable levels of improvements particularly for cellulose degradation in pigs and this is also likely due to the poor or less defined fibre enzyme stability [17, 19]. Clearly, there is a need to further develop more efficacious cellulases to improve efficiency of dietary fiber utilization in commercial swine production.

**Fig. 2:**
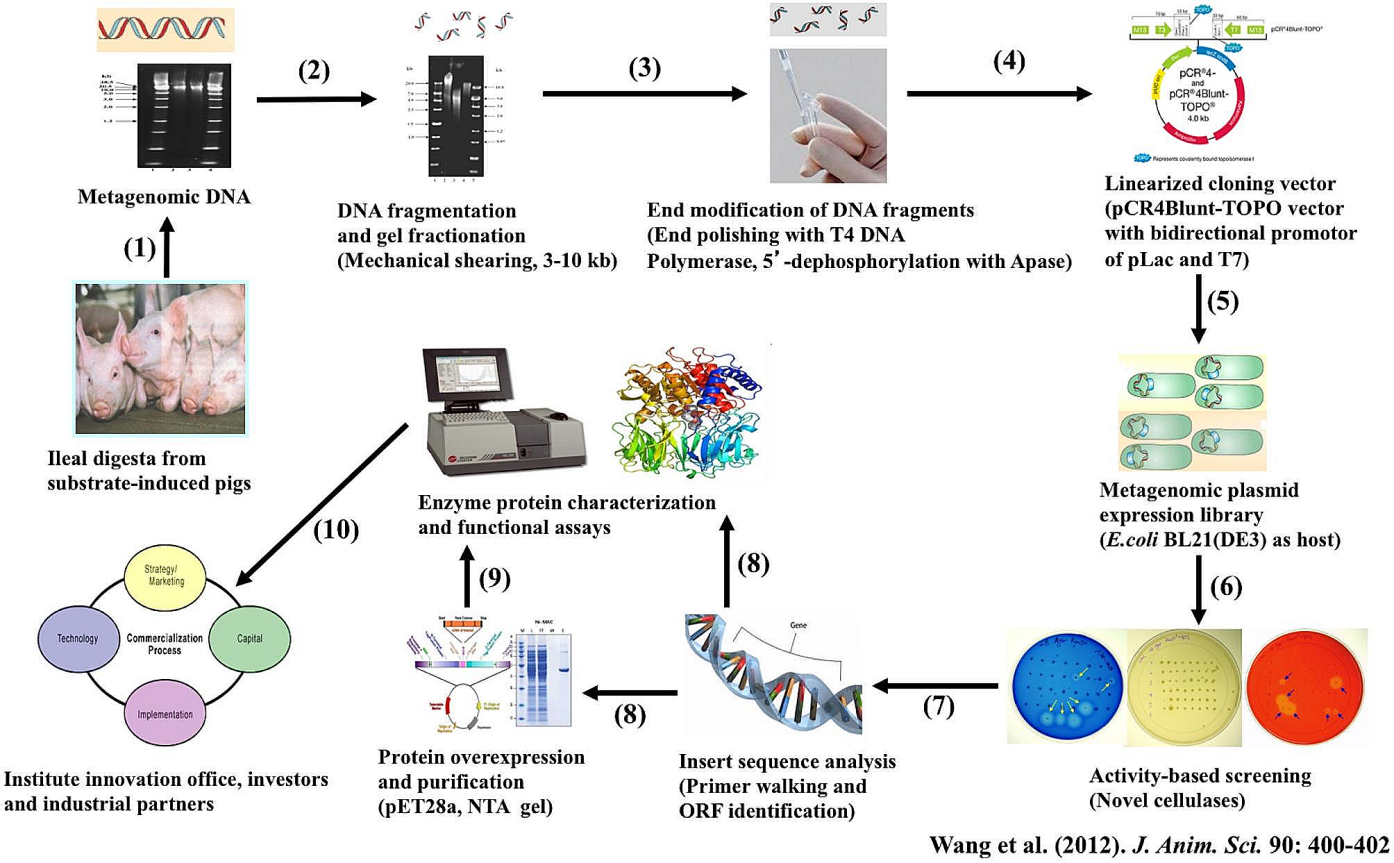
An illustrated road-map of using a metagenomic expression screening library approach in the discovery and characterization of the novel biomass and fibre degradation cellulases from the porcine gut microbiome

When we broadly examined microbial systems that had active fibre biomass degradation activities to search for novel microbial cellulase genes, we were somehow surprised to appreciate the relatively high crystalline cellulose digestibility of 62 and 82%, respectively, measured at the distal ileal and the fecal level in growing pigs when fed a high-fat and animal protein-based diet with 10% Solka-Floc as the sole dietary fibre in our previous study [14]. We were able to further show that the fractional Solka-Floc cellulose degradation rates at the distal ileal (39%/h) and at the fecal (1.9%/h) levels in the growing pigs were much higher than the fractional Solka-Floc cellulose degradation rate (1.2%/h) in the cow rumen; and these comparisons suggested that the porcine gut microbiome would be a much faster turnover microbial eco-system and possess novel genes encoding highly active cellulases [20]. We also knew that majority of the porcine gut symbiotic bacteria were not culturable to mine novel and efficacious target bacterial cellulases, thus we had chosen the metagenomic approaches. The metagenomic sequencing and cataloguing-based approach, as reported by Hess et al. [21], was also not financially feasible for our research program [22]. We had chosen the metagenomic functional expression screening library as a feasible approach, as further illustrated in Fig. 2. We established a metagenomic functional cellulase expression screening library through using the porcine gut metagenomic DNA purified from the distal ileo-cecal digesta of growing pigs fed the Solka-Floc cellulose-based diet for 4 wk [22]. A positive clone harboring the glycosyl hydrolase family-5 (GH5) endocellulase gene, referred to as GH5-p4818Cel5_2A, showed a superb cellulase activity and was effectively screened out of the established porcine hindgut microbial metagenomic expression library [20]. The porcine gut microbial GH5-p4818Cel5_2A endocellulase activity was 307– to 1079–fold higher than the activities of the endocellulases that were characterized from the ruminal *Fibrobacter succinogenes* as reviewed by Fan et al. [2]. And these comparisons are consistent with the above-compared patterns of the fractional Solka-Floc cellulose degradation rates between the pig and the cow.

This porcine gut symbiotic microbial GH5-p4818Cel5_2A processive endocellulase has matched well in several aspects with another unique GH5 processive endoglucanase, referred to as GH5-tCel5A1, that was originated and then truncated from the extremely thermophilic *Thermotoga maritima* [2, 23]. Both GH5-p4818Cel5_2A and GH5-tCel5A1 are characterized as monomodular endocellulases without requiring a carbohydrate-binding domain for the hydrolysis of crystalline substrates and have relatively small molecular weights with an estimated spherical diameter at about or < 4.6 nm [2, 23]. Both GH5-p4818Cel5_2A and GH5-tCel5A1 endocellulases have an optimal pH at the slightly acidic pH and act as processive β-1,4-endoglucanases [2, 23]. The porcine GH5-p4818Cel5_2A is a mesophilic cellulase enzyme, whereas the GH5-tCel5A1 is a thermophilic cellulase enzyme [2, 23]. Both endocellulases are active in hydrolyzing natural crystalline and pre-treated cellulosic substrates and have multifunctionality towards several hemicelluloses including β-glucans, xylan, xylogulcans, mannans, galactomannans and glucomannans [2, 23]. Cheng et al. [7] further showed that both GH5-p4818Cel5_2A and GH5-tCel5A1 endocellulases had similar enzyme activities (0.0090 vs. 0.0080 µmol/min•mg protein) towards the assay crystalline cellulose substrate Avicel at the porcine gut physiological temperature and pH of 6.0. However, both GH5-p4818Cel5_2A and GH5-tCel5A1 endocellulases were susceptible to auto-oxidation by the airborne O_2_ and were unstable under the gastric acidic-pH and proteases conditions [7]. Thus, post-fermentation enzyme processing such as coating and/or site-specific mutagenesis enzyme protein engineering may be further needed in order to further optimize both the GH5-p4818Cel5_2A and GH5-tCel5A1 processive endocellulases for their future commercial applications.

### Current fibre enzymes and emerging endocellulases in swine nutrition and gut microbiome

**Fig. 3:**
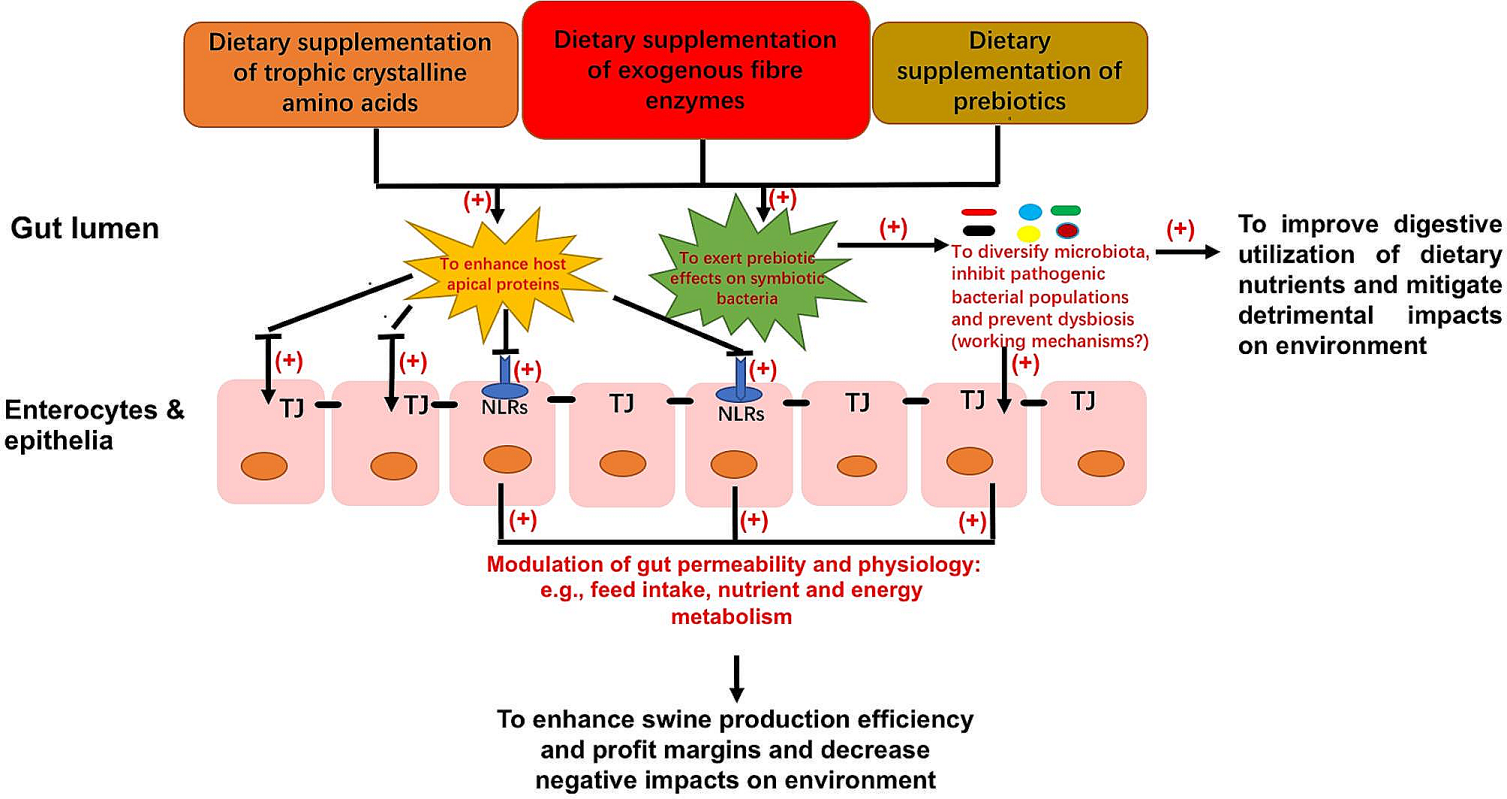
Proposed mechanistic contributions of exogenous fibre degradation microbial enzymes along with prebiotics and trophic amino acids as essential gut modifiers and/or therapeutics in modulation of the porcine gut microbiome, gut permeability and growth performances in adaptation to chronic heat stress with adaption from Fan et al. [6]

It has been well documented that pigs housed under unsanitary conditions and chronic a heat stress environment experience changes in systemic immunity and have decreased growth performance and productivity [8–10, 24]. Pigs and other species of animals and humans under unsanitary housing and chronic heat stress environments typically display changes in intestinal contents of microbial metabolites, gut microbiome and increased gut permeability [10, 24, 25–29]. As shown in Fig. 3, dietary supplementation of trophic AA as well as various probiotics, prebiotics and synbiotics have been proposed and reviewed to improve gut mucosal morphology, optimize gut micro-environment and to maintain gut permeability [10, 30, 31]. Nevertheless, holistic thinking and systemic approaches need to be further taken to curb the human-induced GHG emission and the global warming-effect trend through focusing on developing concrete novel cellulase biotechnologies to improve efficiency of dietary fibre utilization by the porcine gut in swine production and biomass processing for the entire overall bioeconomy sectors.

Through the past decade of multi-disciplinary research efforts, both GH5-p4818Cel5_2A and GH5-tCel5A1 have been discovered, developed and characterized as multifunctional endocellulases and are known to act uniquely as monomodular and processive endocellulases in hydrolyzing dietary fibre and other biomass origins of natural cellulose materials into soluble cello-dextrin and D-glucose as well as degrading various hemicelluloses into hemicellulose-derived oligosaccharides and sugars that have prebiotic effects [2, 7, 20, 22, 23]. According to Gibson and Roberfroid [32], fructooligosaccharides were the only defined original prebiotics back then. The prebiotic list has been further expanded to also include inulin, resistant starch as well as various hemicellulose-derived oligosaccharides and their constituent monosaccharide prebiotic sugars that exert the prebiotic effects via enhancing gut beneficial bacterial proliferation, host immunity and other physiological and health parameters [33, 34]. Although ß-glucans and pectin are the common viscous soluble fibre components in swine feeds, these two viscous soluble fibre components are not regarded as prebiotics in swine nutrition [33]. Hayhoe et al. [10] and Ringseis and Eder [31] reviewed that prebiotics are the effective strategies to prevent gut dysbiosis, optimize gut microbiome and maintain gut permeability in pigs under unsanitary housing and chronic heat stress.

The current technologies to provide the hemicellulose-derived oligosaccharide prebiotics and their constituent monosaccharide prebiotic sugars to swine diets include dietary supplementation of various exogenous microbial cellulases and hemicellulases and through biological processing of biomass materials [33, 35]. We have thoroughly reviewed a total of 49 original efficacy research papers published during 2004–2022 in the area of dietary supplementation of various exogenous microbial cellulases, hemicellulases and pectinases, including 15 papers in the post-weaned pigs [36, 37] and 34 papers in the growing-finishing pigs [38, 39]. One study reported the use of exogenous xylanase originating from the thermophilic *Thermopolyspora flexuos* [40]. Almost all of our other reviewed studies reported the use of their exogenous fibre enzymes with the genes originating from the mesophilic fungal species of *Aspergillus aculeatus*, *Aspergillus niger*, *Aspergillus sulphurous*, *Fusarium verticilloide*, *Trichoderma longibrachiatumi*, *Trichoderma reesei and Talaromyces versatilis* [38, 41–44] as well as the mesophilic bacterial species of *Bacillus subtilis* WL-1 and *Penicillium funiculosum* [45, 46]. Thus, their study test enzymes were supplemented in the mash form but not pelleted diets. Most of our reviewed these papers reported the use of singular or combined hemicellulases of ß-glucanases, ß-mannanase and xylanases [39, 40, 42, 43, 47–50]. Whereas some studies used the combined multienzyme cocktails of cellulases, various hemicellulases and pectinases [38, 51–54] with showing inconsistent response endpoints in vivo efficacy in growth performance, fibre and other nutrient digestibility, intestinal physiological as well as microbial metabolite and population abundances in the post-weaned, growing-finishing pigs and sows [38, 51–54]. The literature reported total tract pectin digestibility was relatively high ranging from 77 to 79%, as indicated by changes in uronic acid digestibility in growing pigs and gestation sows [54]. Dietary supplementation of pectinases along with other multienzymes could improve the total tract pectin digestibility by 4–5% in growing pigs and gestation sows as indicated by changes in the digestibility of uronic acids including the pectin constituent galacturonic acid [54]. Thus, the lack of consistent efficacy endpoint responses in the currently reported exogenous fibre enzymes are likely due to their less defined *in vitro and in vivo* enzyme stability and limited fibre enzyme biochemical property specific to the common swine diets.

Under this context, the afore-discussed recently emerged monomodular and multifunctional processive endocellulases of GH5-p4818Cel5_2A and GH5-tCel5A1 have three potential advantages to become the potent next-generation designer exogenous fibre biocatalysts. First, they are globular and are much smaller in molecular size, thus highly penetrating into biomass pores for their hydrolytic action; second, these two cellulases are multifunctional and each enzyme molecule can catalyze multiple cellulose and hemicellulose substrates without the need to use multiple enzymes to be economical; and third, these two cellulases are well characterized for their limited *in vitro and in vivo* enzyme stability and functional properties for potential further in vivo efficacy application optimization through use of commercially available enzyme coating polymer compounds. Therefore, further research efforts will be needed to enable these cellulase enzymes to emerge as novel biocatalysts for biomass processing to produce the hemicellulose-derived oligosaccharide and/or their constituent monosaccharide sugar prebiotics and as new porcine gut exogenous microbial feed enzyme modifier additives to optimize gut microbiome, maintain gut functionality and enhance growth performance and productivity for pigs through exerting prebiotic effects in adaptation to unsanitary housing conditions and a chronic heat stress environment under the tropical and sub-tropical swine production setting.

## Conclusion

Poor efficiency of dietary fibre utilization is the recognized core issue of the pork production sustainability. Poor dietary fibre utilization not only limits global pork production profit margin but also collectively contributes to significant anaerobic biogenesis and emission of the potent GHG and the global warming effect. Optimization and development of more efficacious next-generation designer multifunctional monomodular processive cellulases will enable these newly emerging biocatalysts to unlock biomass for bio-processing and as exogenous feed enzyme gut modifier additives. We anticipate that these newly developed gut modifier feed enzyme additives can unleash dietary fibre sugar constituents as metabolic fuels and prebiotics to optimize gut microbiome and maintain gut functionality, nutrition, growth performance and productivity in pigs for adaptation to challenged global swine production environmental conditions such as unsanitary housing and chronic heat stress.

## Data Availability

Not applicable.

## Abbreviations

AA: Amino acid
CH_4_: Methane
CO2,: Carbon dioxide
CP: Crude protein
GH5: Glycosyl hydrolase family-5
GHG: Greenhouse gases
N_2_O: Nitrous oxide
